# Oral Cancer: A Historical Review

**DOI:** 10.3390/ijerph17093168

**Published:** 2020-05-02

**Authors:** Francesco Inchingolo, Luigi Santacroce, Andrea Ballini, Skender Topi, Gianna Dipalma, Kastriot Haxhirexha, Lucrezia Bottalico, Ioannis Alexandros Charitos

**Affiliations:** 1 Department of Interdisciplinary Medicine, Section of Dental Medicine, University of Bari “Aldo Moro”, 70124 Bari, Italy; francesco.inchingolo@uniba.it (F.I.); giannadipalma@tiscali.it (G.D.); 2 Ionian Department, Microbiology and Virology Lab., Policlinico University Hospital, University of Bari “Aldo Moro”, 70124 Bari, Italy; 3 Interdepartmental Research Center for Pre-Latin, Latin and Oriental Rights and Culture Studies (CEDICLO), University of Bari, 70121 Bari, Italy; bottalico.lu@gmail.com (L.B.); alexanestesia@hotmail.com (I.A.C.); 4 Department of Biosciences, Biotechnologies and Biopharmaceutics, Campus Universitario “Ernesto Quagliariello” University of Bari “Aldo Moro”, 70125 Bari, Italy; 5 Department of Precision Medicine, University of Campania“Luigi Vanvitelli”, 80138 Naples, Italy; 6 Department of Clinical Disciplines, School of Technical Medical Sciences, University of Elbasan “A. Xhuvani”, 3001 Elbasan, Albania; skender.topi@uniel.edu.al; 7 Surgery, Regional Hospital “X. Kongoli”, 3001 Elbasan, Albania; 8 Department of General Surgery, Medical Faculty, University of Tetovo, 1220 Tetovo, North Macedonia; kastriot.haxhirexha@unite.edu.mk

**Keywords:** oral cancer, oral surgery, oral infections, history of oral surgery, history of medicine

## Abstract

*Aim*: This historical medical literature review aims at understanding the evolution of the medical existence of oral cancer over times, particularly better comprehending if the apparent lower prevalence of this type of cancer in antiquity is a real value due to the absence of modern environmental and lifestyle factors or it is linked to a misinterpretation of ancient foreign terms found in ancient medical texts regarding oral neoplasms. *Methods*: The databases MedLne, PubMed, Web of Science, Elsevier’s EMBASE.com, Cochrane Review, National Library of Greece (Stavros Niarchos Foundation, Athens) and the Library of the School of Health Sciences of the National and Kapodistrian University of Athens (Greece) were extensively searched for relevant studies published during the past century on the history of oral cancer and its treatment from antiquity to modern times, in addition to the WHO website to analyse the latest epidemiological data. In addition, we included historical books on the topic of interest and original sources. *Results*: Historical references reveal that the cradle of the oral oncology was in ancient Egypt, the Asian continent and Greece and cancer management was confined to an approximate surgical practice, in order to remove abnormal masses and avoid bleeding with cauterization. In the Medieval Age, little progress occurred in medicine in general, oral cancers management included. It is only from the Renaissance to modern times that knowledge about its pathophysiological mechanisms and histopathology and its surgical and pharmacological treatment approaches became increasingly deep all over the world, evolving to the actual integrated treatment. Despite the abundant literature exploring oncology in past civilizations, the real prevalence of oral cancer in antiquity is much less known; but a literature analysis cannot exclude a consistent prevalence of this cancer in past populations, probably with a likely lower incidence than today, because many descriptions of its aggressiveness were found in ancient medical texts, but it is still difficult to be sure that each single description of oral masses could be associated to cancer, particularly for what concerns the period before the Middle Ages. *Conclusions*: Modern oncologists and oral surgeons must learn a lot from their historic counterparts in order to avoid past unsuccessful efforts to treatment oral malignancies. Several descriptions of oral cancers in the antiquity that we found let us think that this disease might be linked to mechanisms not strictly dependent on environmental risk factors, and this might guide future research on oral cavity treatments towards strategical cellular and molecular techniques.

## 1. Introduction 

Oral cancer and its symptoms and signs have been observed and described by medicine since ancient times; this review spans from the first approximate descriptions of oral neoplasms by several important physicians and surgeons of ancient past civilizations to the current and emerging approaches for treating them. Oral cancer includes cancers of lip and all subsites of the oral cavity and oropharynx [1]. It represents the 16th most common malignancy and the 15th leading cause of death worldwide, with an incidence of oral cancer (age-adjusted) in the world of four cases per 100,000 people, with a wide variation across the globe which depends on gender, age groups, countries, races and ethnic groups and socio-economic conditions [1,2]. Most differences between the developing world and the Western world are undoubtedly caused by differing population habits, life expectancies, preventive education and the quality of medical records in various countries (poverty, illiteracy, advanced stage at presentation, lack of access to health care, and poor treatment infrastructure) [3,4]. Many physical conditions, environmental and genetic factors are established risk factors for oral cavity cancer [5,6,7,8,9,10].

Thus, while in North America and Europe “high risk” human papillomavirus (HPV) infections are responsible for a growing percentage of oropharyngeal cancers among young people, for other infectious agents this link is still debated (e.g., has been reported that different species belonging to the genus *Candida* produce endogenous nitrosamines from dietary nitrites present in the oral cavity, especially in saliva) [5,6,7,8,9,10]. Its mortality rate remains high, depending above all on the stage of the disease at the time of diagnosis, which is often already advanced. 

Instead, the real prevalence of oral cancer and its mortality worldwide in antiquity is a more obscure field. There is large literature exploring the history of different aspects of oncology in multiple languages and across continents. Despite this, historically speaking, when analysing the medical literature before the 15th century, there is a strange and intriguing lack of references to oral cancers. This review tries to better review the historical medical literature in order to understand if oral cancer’s scarce descriptions could be attributed to a real lower prevalence due to different lifestyle and environmental factors or, simply, its past clinical descriptions and treatments have suffered long subjective interpretations in an antique medical language. A better knowledge of the evolution of oral cancer diagnosis and treatment throughout history should bring a better approach for treating such an affliction and pave the way for future research.

## 2. Materials and Methods 

An extensive search for historical papers and textbooks on the topic of interest was carried out for a narrative review from the early history of oral cancer to date. We included historical papers and reviews by using the search engines of Web of Science, MedLine, PubMed, Google Scholar and Elsevier’s EMBASE.com, with several keywords (Oral cancer; Oral surgery; History of oral surgery), consultation of original documents from Greek historical online archives and treatment guidelines from the WHO website. Due to the uncertainty of the diagnosis of oncologic conditions affecting the head and neck, and of related terms, we preferred use a free style search instead of MeSH. We searched the literature containing medical and/or surgical descriptions of oral cancer management in antiquity, written in English or translated into English from a foreign language (Chinese, Egyptian, Indian, Greek and Latin), in German and Spanish, but also in the polytonic or monotonic, orthography of ancient and modern Greek, in order to refer to the original ancient versions of the texts and better understand its historical and medical meaning, avoiding a secondary reference. Also, for this purpose we have extended the research to the National Library of Greece (Stavros Niarchos Foundation, Athens) and the Library of the School of Health Sciences of the National and Kapodistrian University of Athens (Greece). Abstracts in the original language (followed by English translation) of some of the most representative texts were provided in order to better understand its historical and medical meaning, avoiding a secondary reference, but also to allow other native speakers scholars to formulate their personal hypotheses on terms and items in ancient languages whose interpretations is still debated. 

One hundred and forty-five documents were eligible for the study, dating from 1665 to 2019. Papers were included for their medical and historical relevance about oral cancer diagnosis and treatment throughout the ages, comparing medical knowledge of different medical cultures all over the world. Particularly, we selected documents and books which were best focused on oral cancers detailed description rather than those who talk about general aspects of oncology, original medical texts written by well-known ancient physicians and illustrious surgeons, avoiding as much as possible secondary references. 

Papers with historical relevance investigating oral cancer’s descriptions, prevalence, clinical features, diagnosis and treatment throughout the ages made by ancient physicians were included. Their findings were assimilated, starting with ancient times to conclude with the most recent discoveries about this malignancy’s approach of the twentieth and twenty-first centuries. Furthermore, we selected from literature some papers regarding famous historical figures affected by oral cancer to discuss about how oral cancer management has changed over time.

## 3. Results

### 3.1. Ancient Egyptian Civilization

Ancient Egyptians medicine is one of the oldest practices ever documented. Egyptian medical practice from the late 4th millennium BC was extremely advanced for its time, enough to distinguish it from other civilizations [11], as Herodotus reported ‘‘the country is full of doctors: some of the eyes, some of the head, some of the teeth and some of matters relating to the abdomen and some of internal diseases” [12]. In fact, medicine as a scientific system appeared initially as a ‘Mediterranean phenomenon’ involving the first Egyptian and Greek civilizations. Through the interpretation of Egyptian hieroglyphic inscriptions and papyri, we know that their physicians were highly knowledgeable about medicine, they were able to practice simple non-invasive surgery’s techniques, including dental practices [13], and the art of bone setting in human beings; moreover, they experimented many therapeutic uses of plants extracts and natural substances, including them in an extensive pharmacopoeia. The ancient Egyptians were conscious of the importance of a moderate and balanced diet for a healthy life: their alimentation was based on emmer wheat and barley, oil from the linseed plant, vegetables and fruits. Meat and fish were widely consumed, particularly among the upper classes. They advised patients to avoid foods such as raw fish or other animals considered to be unclean [11,14] 

References to the Egyptian medical and surgical practices from 3000 to 2500 BC have been deciphered in extensive papyri and hieroglyphics found on ancient ruins, particularly in two of the most important Egyptian papyri which are the “*Ebers Papyrus*” and the “*Edwin Smith Papyrus*”. Both Papyri are dated between 1600 and 1550 BC but are believed to contain descriptions originating from 2500 and 3000 BC [15,16,17,18,19]. They are named after the egyptologists who found them in the 1800s. The *Ebers Papyrus* is more focused on medical practice; in fact, it contains 877 prescriptions to cure hundreds of ailments and illnesses, punctuated with magical charms and incantations [4]; in this ancient document, the description of several numerous possibly cancerous lesions is reported, generally translated as a “swelling” or “tumor”; in the *Ebers Papyrus’* final section*,* entitled “*Treatise on Tumours*”, two medical cases (553 and 554) describe cancerous ulcerations of the oral cavity as ‘‘*an eating ulcer on the gum*” with the ancient Egyptian term *“bnwt”.* References [20,21,22] case 857 describes, with the Egyptian term “*ḥnḥnt*” a probable cancerous lesion of the throat, described as a soft swelling in the neck of a man, with fluid vesicles and which was treated by local medications; [20,23] cases 859 (a *ḥnḥnt*) and 861 (a *nḥnt nt rj.t*) could be interpreted as purulent lesions of the throat [21]. The *Edwin Smith Papyrus,* on the other hand, is a textbook concerning surgery, with detailed anatomical observations and the “examination, diagnosis, treatment, and prognosis” of numerous ailments; it reports the first description of a breast cancer [24]; this papyrus suggested medical and surgical management of cancers, including ointments made from animal tissues, vegetables, fruits and minerals, knives, cautery, hooks, drills, forceps, pincers, scales, spoons, saws, incense and arsenic paste for ulcerative tumours. It is thus viewed as a learning manual which probably summarizes medical information from more ancient texts. 

The extensive use of surgery, autopsy and mummification practices gave Egyptians a large knowledge of the body’s anatomy and physiology. Surgery was a common practice among physicians as treatment for physical injuries: there is evidence that oral surgery has been performed as early as the 4th Pharaohs Dynasty (2900–2750 BC) [24,25]. Moreover, they used artificial toes and eyeballs to replace missing body parts [26]. Dentistry was an important profession which dates from the early 3rd millennium BC. All Egyptian remains have sets of teeth in quite poor states because their diet was contaminated by many abrasives (sand left over from grinding grain and bits of rocks in which the way bread was prepared). From 4000 BC to 1000 AD, archaeologists noted a constant decrease in the incidence and severity of worn teeth, probably due to improved grain crushing techniques. Severe dental diseases could even be fatal, as a result of a large infected cyst. In many cases, dental treatments were frustrated and the infected teeth fell out. Some remains show sign of forced tooth removal, probably using opium for treating extreme pain. Replacement teeth have been found, although it is not clear whether they are just post-mortem cosmetics [26,27,28,29]. In historical findings of the Mesopotamian civilization, one of the oldest and earliest human ones and in southwestern Asia, no mentions about cancerous lesions were found [20,30,31].

### 3.2. The Indian Antiquity 

The Sushruta Samhita (Suśrutasaṃhitā, literally “Suśruta’s Compendium”) is an ancient encyclopedic Sanskrit medical text (mid-1st millennium BC), considered one of the most important treatises on medicine and surgery, and one of the most important treatises on this subject which has survived from the ancient Indian world [32]. It is considered the inspiring text of Indian traditional medicine, the “Ayurveda”, written by the Indian surgeon Sushruta. This Hindu text, in its pathology section, is possibly the earliest effort to classify diseases and injuries, through an accurate description of signs and symptoms of different diseases, including body tumors. The ancient terms used for tumors and metastases in this text are “Arbuda” and “Arbudam” [32,33,34]. All the 16th chapter of the encyclopaedia is dedicated to oral pathologies, which are described under the terms of “Mánsaja” referring to lip cancer, “Mahá-Saushira” and “Arvuda” referring to alveolar and palatal cancer, “Alása” for cancer of the tongue’s base and “Adhjihva” for cancer of tongue’s tip, “Rohini, Śataghni and Valása” to describe pharyngeal and hypopharyngeal tumors, “Kaphaja Rohini, Valaya and Giláyu” types for tumors of the post-cricoid and oesophagus, “Svaraghna” for laryngeal tumors. The abundance of detailed descriptions and mentions of oral pathology and the attention of the author focused on these kind of cancer lead us to think how these oropharyngeal diseases and tumors must have been quite common and diffused among Indian people of that time, reflecting a similar condition to the current situation. It is very interesting to note how, in the Sushruta Samhita period, one of the most established risk factors for oral cavity cancer was already documented: the chewing of betel quid’s, a mixture of “Areca catechu” (areca nut), “Catechu” (Acacia catechu) and betel leaf (Piper betel). Sushruta also described many surgical techniques under eight headings: “Chedya” (excision), “Lekhya” (scarification), “Vedhya” (puncturing), “Esya” (exploration), “Ahrya” (extraction), “Vsraya” (evacuation), and “Sivya” (suturing), for a total of 300 surgical procedures, but also all the basic principles of surgery such as planning precision and haemostasis, and various reconstructive procedures for different types of defects [20,35,36].

### 3.3. Ancient Greece and the Roman Period 

From 1750 to 1450 BC, in Greece, the Minoan (Μινωικός) civilization, the first known civilization in Europe, reached its utmost splendour. Crete’s medical knowledge about otorhinolaryngology (*ωτολαρυγγολογικὲ*ς)**, particularly regarding stomatologic (*στοματικές*) pathologies and many other diseases is revealed in many findings displayed in the collection of the National Archaeological Museum of Heraklion in Crete: this ancient collection includes frescoes, idols, sculptures, instruments and different documents [37]. 

Ancient Greek medical authors and practitioners were very interested in understanding cancer’s pathophysiology and in finding surgical techniques for treating cancerous lesions on the skin and mucosal membranes [38,39]. Most of this detailed knowledge was developed through time, beginning mainly from Pythagorean physicians (6th century BC) [40] and later from Hippocrates and the Hellenistic period. Hippocrates of Kos (in Greek *Ἱπποκράτης ὁ Κῷος* 460-370 BC) was one of the most prominent, outstanding figures among the Greek physicians of Classical Greece and in fact of the history of medicine, being considered the ‘‘father of modern medicine’’ and founder of the Hippocratic School of Medicine, which established medicine as a profession for the first time in Greece [41,42,43].

In the “*Ιπποκρατικό Σώμα”* (The *Hippocratic Corpus*) [44], a collection of around 60 ancient Greek medical texts strongly presumed to be associated with his medical theories, Hippocrates dedicated special attention to cancer, reporting multiple references to the management of cancers. Hippocrates and his disciples proposed a rational scientific theory of cancer with the “*humoral theory*” (*θεωρία* των *χυμών*) on its origins, associating it with natural causes and dissociating it from the idea of a religious punishment; particularly, they believed cancer was the result of unfavourable humoral fluxes (*αίμα*=blood, *ξανθή χολή*=yellow bile, *μέλαινα χολή* =black bile and *φλέγμα=* phlegm or mucus) and then caused by an extravascular effusion of these fluids into soft tissues, but also they believed that cancer was strongly linked with excess or lack of food and with old age [45,46]. The origin of the term “cancer” is credited to Hippocrates and to the Hippocratic physicians, who used the Greek terms *kαρκίνος or kαρκίνωμα* (karkinos*/*karkinoma*=cancer)* in order to describe tumours, in reference to the shape and texture of a cancerous lesion in the breast of one of his patients: in fact, the finger-like spreading projections departing from a cancer called to mind the shape of a moving crab, infiltrating tissues with its claws [19].

Hippocrates and his followers proposed surgical techniques and palliative drugs against several form of body’s malignancies, such as pharyngeal carcinoma, that he used to treat, after other therapeutic and dietary approaches, using local surgical excision and cauterization, as he affirmed in one of texts of the Hippocratic corpus, *“Aφορισμούς”* (The Aphorisms; chap.7,87) [47,48], written around 400 BC (see Supplementary Materials):

“…*Those diseases which medicines do not cure, iron cures; those which iron cannot cure, fire cures; and those which fire cannot cure, are to be reckoned wholly incurable*…”

Variant translation: “…*What cannot be cured by medicaments is cured by the knife, what the knife cannot cure is cured with the searing iron, and whatever this cannot cure must be considered incurable*…”.

He used to treat neoplastic lesions with “καυστήρ or καυτήριον” (burner or ardent) from the Greek verb καῦσις= to burn), a surgical tool with a burning and haemostatic function on the growth. The term was then translated into Latin as *cauter* or *cauterium* or *ferrum* or *ferramentum* [49]. On the other hand, in case of hidden tumours, he suggested refraining from any treatment, believing these cancers were incurable. In his anatomical and physiological studies on polyps and their removal, he described the communication between the pharynx and the nasal cavity, the respiratory track and the trachea as the organ that starts from the pharynx, ends in the lungs and is composed of rings connecting breathing to the nose. He suggests the necessity of a deep examination of both nasal cavities and oral cavity in cases of breathing disturbances, secretions, acute and chronic sinusitis and oral pathologies [50,51]. 

From the 1st–2nd century AD, Archigenes of Apamea (Ἀρχιγένης ὁ Aπαμεύς), a Greek physician chiefly influenced by the doctrines of the Pneumatic school (*Πνευματική Σχολή)*, founded in Rome by the Greek physician Athenaeus of Cilicia (Ἀθήναιος, 1th AC), experimented many surgical excisions of cancerous growths. Later, his cancer removal technique was well-described in the texts of the Greek Byzantine physician Oribasius of Pergamum (Oρειβάσιος ὁ Περγαμηνός, 325–403 AD); Archigenes believed that certain types of carcinoma, recognized in an early stage, could be treated just with medicaments, avoiding surgery, but if, nevertheless, tumours were found in an advanced stage, they must necessary be removed surgically because they had lost any connection with the rest of the body; he used the proportionate instrument for the incision of the involved area and the completely removal of the neoplastic mass, after surrounding the cancer’s vessels with a complete ligature and preserving nerve anatomy; to stop haemorrhages, he cauterized the area and tried to stop the bleeding part with a piece of cloth, so he sutured with astringent ingredients the surgical wound and prescribed the application of very cold water to avoid bleeding [39,42,52].

Greek medical knowledge was recognized and appreciated by Romans; in fact, Gaius Julius Cesar (100 BC–44 BC), in the second century BC, introduced a law that granted citizenship to all Greek physicians who would decide to practise their medical art in the Roman Empire; among these were, for example, Arcagatos of Sparta (Ἀρχάγαθος), Asclepiades of Bithinia or Prusa (Ἀσκληπιάδης ὁ Βιθυνός,124 BC–56 AC), Dioscorides Pedanio (Διοσκουρίδης Πεδάνιος,40–90 AC), Areteo di Cappadocia (Ἀρεταῖος ὁ Καππαδόκης, 120–200 AC), Galen of Pergamus (Γαληνός ὁ Περγαμηνός, 130–200 AC) and others [39,53,54].

In the same period, the Roman author Cornelius Aulus Celsus (30-25 BC to 38-50 AC) wrote in Latin his medical encyclopaedia ‘‘*De Medicina*” [55], including all the current medical knowledge of the Roman Empire. He made a distinction between the term “carcinoma”, which he used to describe malignant tumours, and “cancer”, which indicated all tumoral growth. He described both types in the face, ear, lips and nose: “…*Id vitium fit maxime in superioribus partibus, circa faciem, nares, aures, labra*…” [55].

In this important opus, he incorporated documents on several cancers, prescribing treatments ranging from topical remedies to surgical removal. He did not recommend surgical excision as a first-line treatment against these malignancies, but rather as a possibility to restore the patient’s ability to eat normally in the case of lip cancer, using excision and cauterization: “…*in labris vero, si nimium contracta sunt, usui quoque necessario iactura fit, quia minus facile et cibus adsumitur et sermo explicatur…*” [56].

In the 2nd century aC, the Greek physician, surgeon and philosopher Galen of Pergamon in his treatise “*Παρὰ φύσιν ὄγκοι*” (*On tumours against nature*), made a detailed classification of anomalous and unnatural body’s masses, that he attributed to the increased level of black bile coming from the liver in the involved tissue, left aside by the purification process by the spleen; this could appended when the diet was unhealthy and the liver and spleen very weak, producing a large amount of fats and dirty blood. He postulated that neoplasms were due to an excess of black bile, which consolidated in certain tissues such as the lips and the tongue. He introduced the terms *ὄγκος/ὄγκοµa* (ògos/ògoma=volume/swelling) to describe the neoplasms. He suggested removing tumours with an extreme carefully surgical procedure, making incisions around them and with an accurate cauterization and ligature of vessels. He also suggested purifying the blood before surgery with purgative medicaments [57,58,59].

### 3.4. The Middle Ages and the Medical Practices of the Byzantine Empire

There came a time when the Roman emperor Flavio Valerio Aurelio Costantino, or Constantine I the Great (274–337 BC), moved the capital of the Roman Empire from Rome to the ancient Greek city of Byzantium and called it *Nova Roma* or *Constantinople* (Κωνσταντινούπολις, 326 BC, present day Istanbul, Turkey). He allowed the spread of Christianity by promoting it and later in the 4th century AD, the Christian religion became the official religion of the entire Roman empire and it was thus that medicine, after the classical era, met Christian thought [60,61]. A group of brilliant physicians in Byzantium (the Christian Roman empire), the natural successor of the Roman classical age empire, made substantial contributions to the history of otolaryngology and therefore stomatology. They explain accurately in their texts details of new important surgical procedures and therapeutic treatments but, at the same time operation techniques originating from earlier Greek, Hellenistic and Roman period medical texts survived, celebrating the works of the Pythagorean physicians Democides of Croton (Δημοκήδης ὁ Κροτωνιάτης 6th century BC) and Alcmaeon of Croton, who first dealt with the human neurosensory functions in the work *“Περί Φύσεως”* (About Nature), Hippocrates, Praxagoras of Kos (Πραξαγόρας ὁ Κῷος, 4th-3th BC), the Alexandria of Egypt Hellenistic medical school’s (288–300 BC) scholars Herophilus of Chalcedon (Ἡρόφιλος ὁ Χαλκηδών, 330-260 BC) and Erasistratus of Ceos (Ἐρασίστρατος, 304-250 BC), Asklepiades of Bithynia, Dioscorides Pedanius, Rufus of Ephesus (*Ρούφος* ὁ Ἐ*φέσιος*, 98–117 AD), Aretaeus of Cappadocia, Galen and other illustrious Greek physicians. Among the Byzantine doctors we must cite Oribasius of Pergamum, Aetius Amidenus, (*Ἀέτιος* ὁ *Ἀμιδηνός,* 502–575 AD), Alexander Trallianus (*Ἀλέξανδρος ὁ Τραλλιανός*,6th century AD), Paul of Aegina (*Παύλος* ὁ *Ἀιγινήτης*, 625-690 AD), Meletius the Monk (Μελέτιος ὁ Μοναχός, 8th to 9th century AD), Leon Iatrosophist *(Λέων Iατροσοφιστής,* 9th century*)* and Nicholas Myrepsos the Actuarios *(Νικόλαος Μυρεψός* ὁ *Ἀκτουάριος,* 13th century) [37,39,40,60,61,62,63,64,65,66,67,68,69,70].

Byzantine physicians enriched Classical Greek medicine with detailed descriptions of laryngeal and pharyngeal surgery in their Byzantine medical texts, introducing new diagnostic modalities and treatments in dentistry, oral pathologies (aphthae, ulcerative and septic stomatitis, gingivitis, glossitis) but also describing oral cancers on the tongue and the lips [65,66,67,68].

Oribasius of Pergamum provided us a precious source in the history of ancient medicine, his “*Ιατρικαί Συναγωγαί”* (Medical Collections), a massive opus composed by seventy volumes (of which only 25 books have survived) that represent a compilation of the medical knowledge of that time. He reports on the medical practices of many his predecessors, such as the Greek surgeons Antillus (*Ἀ*ντυλλοs, 2nd century BC) and Heliodorus (Hλιόδωροs, 1th AD). In some parts of this medical collection, such as in the 9th volume, *“Σύνοψις προς Ευστάθιον”* (Synopsis ad Eustathium filium), particularly in *Βιβλίον Γ*΄ (Book III) “*Περί ύλης ιατρικής”* (De Materia Medica), *Βιβλίον Ε*΄ and *ΣΤ*΄ (Book V and VI) and the 4th volume “*Προς Ευνάπιον περί ευπορίστων (φαρμάκων)*” (Ad Eunapium libri *IV*), Βιβλίον Δ΄ (Book IV) *“Περί νόσων β΄”* (About Diseases, II), he speaks about the pharmacological and surgical management of different oral cavity diseases, such as swelling of lips and tongue, acrochordons (warts or fibroepithelial polyps) of the lips, nose and ear, cheilitis and stomatitis, tooth decay and gum diseases, while in *Βιβλίον με*΄(Book LX) he refers to surgical treatment of cancerous growths on the lips, ears, nose and eyelids by his predecessors, Rufus of Ephesus and Xenophon (10 BC-54 AD) [66,67,68], In an abstract from this book *“Ἑκ τῶν Ρούφου. Περὶ ἁκροχορδόνων καρκινωμάτων”*, κεφ. 45.11. (From Rufus: about, acrochordons (warts) and carcinomas, chapter 45.11) [67], we can read ancient descriptions of oral malignancies:

“…*Xenophon, in his book on carcinomas, describes some kind of malignant and cancerous growth, writing: it is called carcinoma, and when it arises from any part of the body, it materializes and develops outwards similar to the wart and bulbs, in terms of kind similar to mole, blacker (darker) and tougher (abnormal), more compact and more rounded, resembling more or with a bulb or with the so-called fish eyes or with an unripe berry or with melon or some such other*…

…*it happens that each one of these carcinomas becomes larger in others, smaller in others, and in most are simple, in others torn in two and three parts, and in most remaining the same size, which had in the early years, some getting big, some later, some faster, and even cachexia, and in most giving pain, some not give, and especially to those who would become quite large. And each of these carcinomas develops in other parts of the body, even the one that looks like a wart the same on the lip and on the ear and on the nose*…”

Aetius Amidenus contributed to medical knowledge with the work “*Sixteen Books on Medicine” (Ιατρικά Βιβλία Ἑκκαίδεκα)*, where particularly in the sixteen book, *“Ιατρικών λόγοι ΙΣΤ*΄ *”(Medicinales Logoi exkaidekatos, XVI*), *Βιβλίον H΄* (Book VII), he talked about several oral diseases and their specific treatment and surgical approaches. He suggested the use of a plant, *Dracunculus vulgaris* (previously also reported by Pliny and Dioscorides), as a preventive remedy against some cancers, such as those of the oropharyngeal and laryngeal tract “*…δρακόντιον…καρκίνους…εκτήκειν πεπίστευται*” (We think that Dracunculus could destroy cancer) [59,63,65,68,69].

Alexander Trallianus, in the *“Βιβλία ιατρικά δύο και δέκα”* (*Libri duodecim de Re medica*), in the *Βιβλίον Γ΄* (Book III), wrote about surgical anatomy and treatment (either local treatments or by *φλεβοτομία* = phlebectomy) of pathologies of the salivary glands, especially the parotid, such as inflammatory diseases and tumours [70,71,72,73].

Paul of Aegina, the last physician of the Hellenistic Alexandria of Egypt medical school, wrote the medical compendium “*Πραγματείας ιατρικής βιβλία επτά*” (*Epitomae medicae libri septem)* based on the Hippocratic and Oribasius medical tradition. In *Βιβλίον A΄* (Book I), *Βιβλίον Γ*΄ (Book III) and *Βιβλίον ΣΤ΄*(Book VI), he described several oral diseases and their surgical management, both with phlebotomy and with the use of specific forceps and lancets to grasp tissues while cauterizing to avoid bleeding during surgery. Paul of Aegina was very appreciated in the Arab world; Ḥunayn ibn Isḥāq (808-873 AD), physician and philosopher, was one of his most important translators, but also by western Europeans physicians who translated the Arabic medical texts of the Persian Abulcasis (Abū l-Qāsim Khalaf ibn, 936-1013 AD) and the Persian Abū Bakr Muhammad ibn Zakariyyā al-Rāzī (854–925 AD); in fact, the medical scholars found many references to Paul’s work after they translated Arab texts; the Greek physician, then, became also appreciated among the personalities of famous medieval medical schools of Salerno (*Schola Medica Salernitana*, Italy) and Montpellier (France); his treatise, *Epitomae medicae libri septem*, in the 6th book “*Κατά χειρουργίαν Άπαντα*” (Everything on surgery) was considered an important practical guide for surgeons from the 7th to the 16th century AD, translated into Latin at least three times during the 16th century [67,74,75,76,77].

Meletius the Monk, in the work *“Περί της του ανθρώπου κατασκευής”* (*De humani corporis*), in the section *“Περί Στόματος”* (*On mouth*), Βιβλίον Β΄, κεφ. I΄ (Book II, chapter X), describes in detail the anatomy of the tongue, teeth, gums, tonsils, uvula, palate, larynx, trachea and epiglottis. His work inspired the medical career of Leon Iatrosophist, who lived in the time of Emperor Theophilus (Θεόφιλος, 800-842 AD), Βιβλίον Δ΄ (Book IV) of the “*Σύνοψις ιατρικής” (Medical Synopsis or Medicine Summary*) deals with causes and the treatment of oral pathologies, describing their pharmaceutical management such as mucosal medications, gargles and surgical therapy [61,66,67,78,79].

During the 13th century, Nikolaus Mirepsus the Actuarios wrote a collection of 2656 pharmacological prescriptions, “*Μέγα Δυναμερόν*” (Great Dynameron), consisting of 48 chapters, later translated into Latin as “*Medicamentorum Opus”* or *“Codex Medicamentarius*” [80]. One of this prescriptions describes the *ξηρίον* preparation (*Xirion=*dry powder), composed by a mixture of several ingredient of animal and plant origin, to prepare in different concentrations for the cure of different pathologies; for example, for dyspnea he recommended *Theodore’s xirion (*Θεοδώρου* ξηρίον),* a fragrant resin composed by a mixture of withered roses, grapes and myrrh), while for oral cancer he prescribed a mixture of chopped seashells, burned dates and *Piper nigrum* or *P. longum* or *P. album* (*πεπέρεως=piper*) with roots of nut trees. For the treatment of rhino-pharyngeal cancer [59,60,68,80], he wrote (see Supplementary Materials):

“…*xirion beneficial for the bad smell of the nose... …and for the carcinomas; contains eggshell, almond peel, roasted nutmeg, roasted dates*…”

Generally, Byzantine oral disease therapy was characterized by various chemical mixtures of minerals, metals with ingredients of plant and animal origin, as local remedies, inhalations and steam baths. Some of these ingredients were *Quercus robur L*., *Quercus petrea (Matt.) Liebl., Quercus pubescens Willd., Calendula officinalis L, Rosa gallica L., Rosa centifolia L., Rosa damascena Mill*. (for mouth and throat diseases), castorion (*Καστόριον*, an oily extract with a characteristic smell derived from the glandular follicles of *Castor fiber*, a species from Europe and Asia), ammonium, clay (kεραμίτις) and others. Sometimes they used antidotes, as the *θηριακά φάρμακα (*theriac drugs) because they believed intoxications could cause cancer [59,68,81].

Byzantine physicians became increasingly specialized in different medical fields, both from a clinical and a surgical point of view. One of the specialties was the botanist doctor, probably inspired by the ancient herbalist scholars, called *Pιζοτόμοι* (Rizotomi) by Galen. Galen named *Pharmacheis (φαρμακείς), Pharmacopolae (φαρμακοπώλεις), Myropolae (Μυροπώλες)* and *Migmatopolae* (μειγματοπώλεις) the traders of medicinal herbs. The introduction of analgesic and hypnotic plant extracts, such as the *mandrake* roots, *Papaver somniferum* L. (Μήκων η υπνοφόρος), *Papaver rhoeas L. (*Άγρια μήκων) and *Hyoscyamus* helped many surgical improvements. These anesthetic techniques were already described by the physician and pharmacologist Dioscorides Pedanius in the “*Περί ύλης ιατρικής” (De Materia medica)^81^*. We must mention that, Herodotus (484-410 BC), in *“Ἱστορίαι”* (Histories), mentioned 65 plants and aromatic essences which were commercialized by Phoenicians and other civilizations in Greece, such as *Styrax officinalis* L., that were soon after introduced in the Greek pharmacopeia for medical purposes, as antiseptic and healing agents [12,43,57,59,81,82]. 

Byzantine diagnostic and therapeutic procedures took place in the monastic centers of the Byzantine Empire, called Ξενώνες (Xenones=Hostels). Those institutions, precursors of modern hospitals, were built next to monasteries, following the lead of ancient Asklepieions. The Imperial church (nowadays the Eastern Orthodox Church), with his Patriarch (*Πατριάρχης*, called in present day *His Most Divine All-Holiness the Archbishop of Constantinople, New Rome, and Ecumenical Patriarch*) in accordance with the Emperor, enacted reforms to enforce public healthcare structures and to implement welfare measures for altruistic and charitable purposes, in order to sustain poor people and, in general, the lower ranks of society. We must emphasize that Princess Anna Komnene (Ἄννα Κομνηνή, 1083-1153 AD) was a doctor and hospital administrator like Emperor Manuele I Komnenos (Μανουήλ A’ Κομνηνός, 1143–1180 AD) who had studied and practiced medicine. The most important institutions created in that period were Orphanages, Houses for the Poor, Hostels as Hospitals and Lobbies [59,61,63,83,84]. One of the most important hospitals was the “*Pantocrator Xenon”* (*Ξενών Παντοκράτορος*) founded in 1136 AD in Constantinople, by the Emperor John II Komnenos (Ίωάννης Βʹ Κομνηνός, 1087–1143 AD), next to a monastery of the same name. Management rules and health policy of these structures were governed and regulated by the Emperor himself with *τυπικόv* (typikon=formal rules). Physicians employed in these hospitals were called *αρχὶατρος (arhiatros)*. Later the term Aktouarios (*Ἀ*κτουάριος) reserved for eminent doctors linked to the Imperial court was also applied. The hospital had various wards and medical and nursing staff, and also women practiced medical professions. Empire University of Constantinople, the Magnaura Palace University (*Πανδιδακτήριον της Μαγναύρας)* was founded in 425 AD by the Emperor Theodosius II (401-450 AD); it was the first university instituted in Europe. Professors of medicine were *Ιατροσοφιστές* (Iatrosofistes). Medical students, after several theoretical and practical examinations, could practice medicine on patients. Thereafter, medicine as developed through Roman and Byzantium to finally influence European medicine and later the rest of the Western world [85,86,87,88,89,90,91].

### 3.5. The Renaissance and the Medical Knowledge

The following centuries were quiescent for otolaryngology. During the Western Europe Middle Ages, there was little progress regarding oral cancer medical treatments and surgery, which remained the same as in the past because of religious prohibitions of anatomical dissection and surgical operations, which worsened after the Catholic church interdicted bloodshed and surgery in the 13th century; in fact, before the 16th century, medical literature on oral cancer’s treatment is very imprecise and scarce, and the few descriptions of oral cancers describe how to remove them surgically avoiding bleeding and infectious complications while diagnosis and treatment of larynx and hypopharynx tumours remained obfuscated for a long time as well as their pathophysiology. It is only with the cultural and scientific progress of the Renaissance period and the increasing knowledge on human anatomy that even more deep and relevant observations on surgical oral cancer treatment appeared. In fact, many improvements in medicine and oncology were facilitated by several systematic dissection studies of normal and pathologic anatomy, such as those of Andreas Vesalius (1514-1564), a Flemish physician and anatomist considered founder of modern human anatomy and the author of one of the most influential books on this subject, called “*De humani corporis fabrica*” (*On the Human Body’s Factory*) [92].

One of the most important events involved in the raise of oral cancers incidence was the introduction of tobacco in the Western World in the 16th century. Before the discovery of tobacco smoking as a primary cause of cancer, it was used for medicinal and recreational purposes. Despite many efforts to discourage its consumption, for example a heavy tax laws imposed by King James I in England to avoid its production and consumption, tobacco became very popular in all classes of society and its use and abuse consistently grew [28,93].

The first lip cancer treatment was attempted, in 1650, by Richard Wiseman. In his great opus entitled “*Eight chirurgical treatises on these following heads*…”, composed of two volumes and dedicated to his Majesty Charles II, King of Great-Britain, France and Ireland, he wrote about the surgical treatment of tumours, ulcers, anal diseases, fractures and luxation, but also on infectious diseases such as syphilis [94]. In an abstract from the section “on cancer” [95] he wrote (see Supplementary Materials):

“… *another person of about fifty years of age, having been long diseased with a cancer on the left side of his Tongue, staid in the Country till it had corrupted one half from the root to the tip of it, as also the Ranula and Salivals of that side, as well the external as internal parts. Then being at a lots what to do, he came up to London recommended to Dr. Walter Needham for Cure who, feeing his case so deplorable, advised him to consult others…At a consultation we proposed to palliate the Disease, but he declared to us that he came to Town with resolution to have the Cure attempted, tho’ he died under it. We endeavoured to dissuade him from it, but he persisted earnestly in the having it attempted: to which we agreed to cut off that part of his Tongue, and to cauterize the fordid Ulcer that lay on the side of his Mouth between that Jaw and his Tongue. To which purpose we presently sent for some actual Cautery, and in the presence of that Company I put into his Mouth a Raspatory, and, fixing it between the root of his Tongue, and edge of that Tonsil, pulled away the corrupt Flesh; and then with Olive-Cauteries burnt that to a Crust. Having, as we supposed, consumed the Cancer there, I passed a Probe with a Ligasure into the Ulcer under the Tongue, and brought it out above thro’ a Tubercle, then pulling his Tongue forward, I cut off the cancerated part as it lay, according to its length, from the rip to the root of the Tongue; and after I had permitted it a while to bleed, I cauterized it.”*

(Note: The original texts are reported in Supplementary Materials)

Despite this surgical treatment, his patient reportedly died soon after. In his treatise he also described several cases of different patients with cancers of the tongue, lip, and cheek cancers, talking about what he did as a surgical treatment, the surgical instruments he used, such as cautery and the results [95,96]. In this period, surgeons were very scared and reluctant to perform oral surgery, due to the serious rick of massive uncontrolled bleeding, but also because of the strong disfiguration of the face that the complete cancer removal required. The first complete glossectomy to remove a tumor of the tongue was attempted by the Professor of Surgery Pietro de Marchetti (1589-1673), in 1664 at the University of Padua (Italy). He described the operation in the “*Observationum Medico-Chirurgicarum Sylloge*” [97], describing how he could control bleeding with cauterization [98].

In the following centuries, this kind of surgery developed very slowly because of the risk of infections and difficulties due to the lack of anaesthesia, airway management and risk of haemorrhage. Many new techniques and surgical instruments were invented to avoid haemorrhage during surgery; for example, Antoine Louis (1723-1792) introduced the technique of cut off the blood supply to the tumor with ligature of its vessels, Home made a ring of sutures around the root of the tongue to avoid haemorrhage during glossectomy and in the post-operative period [98].

From the 17th-19th century, cancer was believed to be an infectious transmissible disease; basically, every microorganism that could be isolated from cancerous tissues was considered as a possible cause of the cancer’s development and transmission; the first cancer hospital founded in Rheims, France, by Jean Godinot, was force to move in 1740 to the outskirts of the city, because it was thought that recovered patients inside might transmit the infection to rest of the population [94].

Moreover, the opinion of two prominent seventeenth-century clinicians, Zacutus Lusitanus and Daniel Sennert, spread the contagious theory of oral cancer, because it frequently appeared in the oral cavity as an ulcerate lesion, very similar to other ulcerative conditions of that time, such as syphilis. Zacutus Lusitanus, for example, was so convinced that syphilitic and other infectious disease’s ulcerations were cancerous that he affirmed, in his medical texts, that three boys were attacked with cancer after they occupied for a long time the same bed of their mother, who was affected by the same disease, obviously confusing the cancer with syphilitic lesions which are very similar. For this reason, the term “*chancre*” continues to be used to describe syphilitic sores painless ulcers which most commonly appears on genitals and inside the oral cavity during the primary stage of the infectious disease [94,99,100].

### 3.6. Oral Cancer at 19th Century 

Most of our current knowledge on oral oncology, including diagnostic methods and treatment management, has been developed during the 19th and 20th centuries. Until the mid-19th century, oral oncology continued to apply the same primitive theories on cancer and the same surgical techniques based on the simple removal of what appeared relatively superficial, visible and clearly reachable to local treatment. Oral cancers which were most frequently treated were those located on the surrounding skin or those on the mucosal surfaces of the tongue, gums and the palate. The introduction of general anaesthesia in 1846 was the key event which allowed increasing cancer excisions with the development of many surgical accesses for oral cancer, including sectioning of the lip and mandible by Bernhard von Langenbeck (1810 to 1887) and his colleague Theodor Billroth (1829 to 1894), a submandibular access for tongue tumors in order to tie the lingual artery, avoiding haemorrhages, attempted on 120 patients by Theodor Kocher (1841–1917) [101,102]. 

The 19th century saw further advances through the beginning of the microscopic era and the histopathological evaluation of tumours by Johannes Peter Müller (1801–1858) and the microbiologist Alfred François Donnè (1801–1878). The earliest microscopical evaluation technique was largely defective and poor, so they did not recognize its great potential neither in helping the surgeon in the tumoral accurate removal nor in the histological diagnosis of cancer. Prominent personalities in the histopathology field were Rudolph Virchow (1821–1902), who wrongly believed cancers derived from connective tissue and emerged in the epithelium above, and Karl Thiersch and Wilhelm Waldeyer who, on the other hand, proved in 1865 the origin of cancer from the epithelial surfaces and the subsequent invasion of the stroma. However, despite these steps forward in the comprehension of cancer’s origins, acceptance of histological examination of biopsies was slow and microscope was not soon accepted as an important instrument in cancer’s diagnosis. Just few surgeons, such as von Langenbeck and Billroth, considered surgical biopsies as an essential helping factor during the cancerous complete removal [103].

Simon-Emmanuel Duplay (1836–1924), Professor of Clinical Surgery, was an illustrious personality of the French clinic-pathological school and his studies on cancer started the modern period in oncology. He founded an “anti-cancer league” together with his colleagues of the Faculty of Medicine in Paris as Maurice Cazin (1863–1933), Paul Reclus (1847–1914), Julien Brault (1862–1916) and Ellie Metchnikov (1845–1916). Their great project was to amalgamate their medical knowledge to improve the scientific research on cancer. Soon after, he joined all the researches of himself and his contemporaries in a medical text, written in 1903 with Cazin, entitled “Les tumeurs” (the tumours) [104]. In this text, he talked about cancer as ‘‘*a mass constituted of newly developed tissue, tending to persist or increase’’*, distinguishing benign lesions from malignant ones, and clearly affirmed the association between oral cancer, tobacco and poor nutrition. Furthermore, they identify “leucoplakia”, a firmly attached white patch on the mucous membrane of the oral cavity, as an important risk factor for cancer development [104].

At the end of the 19th century, two important American personalities suffered from oral cancer: Hiram Ulysses Grant (1822–1885), the 18th President of the United States, and Grover Cleveland (1837–1908), first 22nd and then 24th president of the United States, in 1893. Grant was known to be a heavy cigar smoker and drinker for many years. He developed a a right tonsillar pillar growth and a clinically positive node in 1884; when he consulted his doctor, the surgeon George Shrady, he made a biopsy of the tonsillar growth, analysing it under the microscope, but his cancer was already untreatable; to fight cancerous pain, his doctor prescribed smoking cessation and topical application of cocaine hydrochloride solution to the cancerous area; he suffered terribly, despite several injections of brandy and morphine for pain control before he finally died of cancer in 1885 [105,106].

Some years later, President Grover Cleveland was another heavy cigar smoker and drinker; one day, he manifested a swelling on the roof of his mouth and noticed a roughness on his hard palate; after several weeks, when doctors examined the lesions, which appeared ulcerative and cauliflower-like, the diagnosis was oral cancer. Cleveland sustained a secret intra-oral partial maxillectomy (left upper jaw), performed on board his yacht, temporary converted in a surgery room. The tissue breach was later restored with a maxillary obturator, a rubber prosthesis. His personal physician sent a biopsy to the Army Medical Museum for a very confidential examination, which confirmed an epithelial malignancy, precisely a verrucous carcinoma. The president did not want to be remembered for his cancer by people, so he tried to hide his cancer from the media. In fact, his dentist explained to journalists that he had “some dental work done and also is suffering from rheumatism”. He died 16 years after the first surgery [105,106,107].

Sigmund Freud (1856–1939), Austrian neurologist and father of psychoanalysis, was another illustrious personality who suffered from oral cancer. He was a heavy smoker (reportedly more than 20 cigars per day) and this probably led to his heart and respiratory problems and, then, to his cancer. In the late 30s, he began experiencing chest pains, shortness of breath and heart problems but he never gave up smoking. In 1923, when he was 67 years old, Freud fell victim to cancer of the palate. His first surgery lead to severe post-operative haemorrhage and an incomplete excision. During the sixteen remaining years of his life, he underwent an endless series of mouth and jaw operations for cancer that forced him, during the advanced stages of his long disease to use a special prosthesis to cover the defect in his palate. He called it “the monster”. His jaw had by then been entirely removed and multiple prosthesis were required to reconstruct the destructive effects of the tumour; he suffered constant pain for the cancerous nerve roots involvement, scar formation and radiotherapy effects, could not speak, chew or swallow. Despite this, he continued highly smoking every day to the very end of his life in 1939 at the age of eighty-three [106,108].

Henry Trentham Butlin (1845–1912) was one of the first head and neck surgeons of the modern period and an illustrious figure in oncology and, particularly, in oral cancer surgery. In his book *Diseases of the Tongue* [109], written in 1885, he clearly defined how to approach surgery of the lips and of the tongue; he showed that, during partial glossectomy, the prophylactic supra-omo-hyoid neck dissection through the ‘‘Kocher’’ incision (Y-shaped incision) [110] could improve patient prognosis and survival in many cases [111,112]. He can be considered as the father of the modern head and neck surgery, specifically regarding the conception of an even more radical dissection of the primary tumour “*en bloc*” with surroundings cervical nodes [112]. He gave a strong contribution also to the palliative surgery’s field in case of pain for incurable cancers in advanced state and side effects such as hyper-salivation throughout a lingual nerve section and the use of iodoform powder in a very concentrated form. For his successful researches and surgical results, he was designated President of the Royal College of Surgeons in 1909 and the British Medical Association in 1910 [112].

### 3.7. Oral Cancer at the Modern Age (20th Century)

The history of oral cancers’ medical and surgical development continues in the 20th century, with advances in neck dissections and reconstructive surgery, but also in relevant non-surgical options for complete oral cancer management. The involvement of lymph nodes in cancer, recorded as early as 1790, was used in the past as an indicator for incurability [113,114]. William Stuart Halsted (1852–1922), in the late 19th century, used the concept of lymphatic spread of primary tumour’s cells and showed that radical resection with “*en bloc*” node dissection could bring to a 6% reduction in recurrence rates [115]. 

George Washington Crile (1864–1943), in 1905 and 1906, published two papers describing a systematic approach of “en bloc” dissections, which included the sternocleidomastoid muscle, the spinal accessory nerve, and the internal jugular vein and all lymphatics, from levels I-V) and the results of more than 250 operations [116,117]. We must mention that, in 1902, Polya and von Navratil, two German surgeons, first described lymphatic drainage from oral sites, concluding that enlarged nodes could contain occult metastases and should be removed along with the original cancer [118]. Their observations and studies were not translated in English, so the “*en bloc*” neck dissection technique only bears Crile’s name. 

From 1938 and during the following 20 years, Hayes Martin, Chief of Head and Neck Service at Memorial Hospital for Cancer and Allied Diseases in New York, along with his team carried out 1,450 radical neck dissections on patients with oral cancers and cervical metastases, with increasingly improved results and popularity of this surgical technique [119]. Despite this, the great morbidity associated with a so radical dissection led to the development of more conservative techniques. Osvaldo Suarez (1912–1972) was an Argentinean surgeon considered ‘‘the father of functional or modified neck dissection’’ because he experimented, in 1963, with a technique to preserve the accessory nerves and their functions with similar results [120].

In the first mid-90s, radiotherapy was also used to treat oral cancers and its nodes dissemination, trying to avoid the devastating effects of extensive surgery, but the survival rates were poor [121]. Further surgical developments were an even more selective dissection of nodes, introduced by the MD Anderson group in Texas [122] based on the metastatic risk from the primary tumour location. In 1990 Shah et al. [123] demonstrated that the risk of metastasis in lymph nodes of 1,801 patients in levels IV and V was only 9% and 2%, so they concluded that their radical removal in not indispensable in N0 necks. Two decades later, in 2002, the American Head and Neck Society standardized neck dissection terminology and technique, dividing neck nodes in a 6-level classification system [124].

The health benefits of super-selective node dissection in patients with early tumours is what current research is trying to achieve, through the sentinel lymph node biopsy, considering metastatic spread mainly to the lymphatics closest to the primary growth. In modern practice, surgery remains the main treatment for oral cancer, while external radiotherapy and/or chemotherapy can be used as an adjuvant treatment to primary surgery, as main treatment in patients in whom surgery is not recommended and as a palliative treatment in the advanced stages. Particularly, intensity-modulated radiotherapy is the technique to reduce the dose of radiation on salivary glands and mandible, in order to avoid xerostomia and osteonecrosis. Actually, there is no evidence that conventional chemotherapy improves survival rates in these patients, while some results can be observed by using new molecules (cetuximab, epidermal growth factor monoclonal antibody) combined with adjuvant radiotherapy in patients with positive tumour primary margins and extracapsular lymph node spread [125,126,127].

For what concerns reconstructive surgery, modern strategies now available offer the possibility to fill tissues defect by creating a computer tree-dimensional model of bony microvascular free flaps with precise fit [127] and osteo-integrated implants for efficient oral and dental rehabilitation in selected cases [128,129,130,131,132]. The future of oral cancer management will likely include extensive genetic testing of patients to allow adjusted focused treatments, the use of stem cell technology to ‘‘grow’’ compatible organs that could fill tissue gaps without the necessity for immunosuppression [133,134,135,136,137]. Today, despite the advances in medicine and surgical resection in improving temporary patients’ quality of life, over half of oral cancers are in a too advanced stage: the result is that prognosis of oral cancer has not changed much since first mid-90s attempts to save the life of patients with stage III and IV at diagnosis. Extensive neck dissection is still the most important weapon against oral cancer, and the clinical presence of nodes continues to be a fundamental prognostic factor. All major historical events regarding the historical evolution of oral cancer management and treatment are reported and summarized in Table 1.

Currently, while many environmental agents and physical conditions are established risk factors [5,6,7,8,9,10] for oral cancer (Table 2 and Table 3), so they can be easily fought with prevention campaigns (for example against tobacco and alcohol abuse, improving social-economic status, hygienic conditions and adequate sexual behaviors), contemporary physicians and oral surgeons must investigate and learn more about those conditions whose role on oral cancer pathogenesis is still mysterious and unsearched, such as genetic polymorphisms, thinking one step further to possible future oral cancer’s management and strategical cellular and molecular techniques against this malignancy, always considering their historic counterparts, in order to avoid past unsuccessful efforts against this kind of tumour [138,139,140,141,142,143,144,145].

### 3.8. From the Management across Centuries to Future Insights

Globally, as reported from the International Agency for Research on Cancer (IARC), the specialized cancer agency of the World Health Organization (WHO), oral cancer occurs more often in individuals from lower and middle income countries in about 355,000 people and resulted in 177,000 deaths in 2018: of these 355,000, about 246,000 were males and 108,000 females [1,146]. 

During ancient times, the advised treatment was a poultice involving cinnamon, honey, and oil, or in management of pain, arsenic paste or zinc oxide were prescribed. Owing to the nature of hieroglyphic interpretation, there also is considerable disagreement over meanings, rendering the accuracy of the literature questionable [28].

Over the centuries and in particular within the 20th century till today, various specific and non-specific approaches have been introduced that could predict the malignant transformation of oral cancers and to address to novel therapeutic methodologies (precision medicine); however, detail information on these approaches in a concise manner is lacking. Moreover, their use on daily clinical basis still remains questionable [147,148]. With continuous research in the field of cytology, biochemistry, molecular biology, translational medicine and genomics, several contemporary biomarkers and therapies have been discovered that are not yet foregrounded and proved to be more promising than those used conventionally [147,148,149,150,151,152,153,154,155].

A great deal of research is being done to learn what DNA/RNA changes cause the cells of the oral cavity to become cancerous [150]. Gross chromosomal alterations (polysomy, aneuploidy) and specific gene aberrations such as amplifications, deletions, point mutations combined or not with epigenetic ones (promoter methylations and microRNAs (miRNAs) deregulations) are responsible for the progressive transformation of normal squamous epithelia to the corresponding malignant. In most part of oral cancers cases, critical genes, such as TP53, FAT1, NOTCH1, CASP8, and CDKN2A (p16INK4A) and PI3K mutations are found to be inactivated, leading to an overactivated cell cycle correlated to carcinogenetic process [147,153,154]. Moreover, circulating miRNAs, seems to be a useful biomarker to develop preventive strategies [154]. 

Some texts, written in Latin prose, documents several cancers and advises an assortment of treatments, from topical pastes for superficial cancers to surgical excision for hidden and oral, head and neck cancers [28].

Surgery plays a key role, both at early stages as well as in locally advanced or recurrent disease. Fortunately, over centuries, advances in anatomy led to enlightenment regarding surgery, including for head and neck cancer. From ancient age till 19th century, lack of anaesthesia and antiseptic methodology, airway management and risk of haemorrhage make every surgical approach very hazardous for patient’s health. Chemotherapy and radiotherapy in the management of oral cancer was explored in the 20th century [28,138].

Most patients with oral cancers initially present with locally advanced disease, which often requires a multidisciplinary approach involving surgery, radiation, and/or chemotherapy (5-fluorouracil, carboplatin or cisplatin, and taxol-based therapies and irinotecan in the most advanced cases) and recently monoclonal antibody targeted against epidermal growth factor receptor (EGFR). The frequent activation of the PI3K/mTOR pathway in oral cancers and its cancer-driving role may represent a vulnerability that can be targeted therapeutically. This pathway dependence is also being investigated clinically in multiple trials using direct PI3K and/or mTOR inhibitors in oral cavity, as well as by the use of metformin, which blocks mTOR indirectly, for oral cavity tumors prevention in patients with potential premalignant lesions [154,155].

Preliminary data indicates that addition of hyperbaric oxygen (HBO_2_) therapy to chemo-radiation standard of care is technically feasible, well tolerated and safe has is well-know that HBO2 already played a prominent role in both the prevention and treatment of mandibular osteoradionecrosis [156].

Finally, key emerging mechanism of tumor immunosuppression involves T-cell exhaustion. Tumor-associated macrophages (TAMs), derived from inflammatory monocyte, play a critical role in regulating tumor progression. Generally, TAMs promote tumor progression and suppress immune response via both innate and adaptive immune mechanisms. However, as the double-blade sword, TAMs retain the potential pro-inflammatory ability to inhibit tumor progression. By depleting the immunosuppressive function or evoking anti-tumor ability, therapeutic strategies targeting TAMs show promising preclinical and clinical effects. Now, macrophage-centered therapeutic approaches such as anti–PD-1 T-cell, targeted therapeutics that can reactivate antitumor T-cell responses, are entering the clinical arena [154,157].

## 4. Discussion

In the light of all these studies on oral cancer in the antiquity, Egyptian, Indian, Greek and Roman texts mention general information on many different types of tumours, but without specifying the distinguishing and peculiar characteristics and behaviour of each subtype of neoplasm. Early descriptions of oral neoplasms are represented by oral destructive masses that they tried to remove with non-selective surgical techniques. Before excluding a more or less similar prevalence of oral cancer in the past civilization as nowadays, we should be sure that what it is indicated as a specific pathological condition in ancient medical documents really was what we know today with the same term! 

Oral cancer’s global increase has largely been attributed to modern lifestyle and carcinogenic environmentally-related factors such as smoking, diet and pollution; these factors have contributed to increasing risk of cancer as well as a longer life expectancy and gene heredity. On the other hand, the question we have not bothered to ask yet is whether past human society lower rates of cancer compared to those seen among modern civilizations or its apparent lower incidence and prevalence in past human population can be related to an erroneous identification and translation of documents related to this disease? In fact, there is another possible theory to explain of the lack of a wide literature on this cancer: ancient texts are, obviously, written using an antique language style, often very far from the modern one, and this leads to subjective interpretations and mistranslation by each single translator, particularly considering the specificity of medical terminology. Moreover, it is very problematic to understand if a specific term, often ambiguous, was really related to that particular disease which is actually well described with a modern precise scientific nomenclature, distinguishing in ancient literature cancerous and non-cancerous etiologies of such conditions; for example, we cannot easily distinguish if an ancient hieroglyph representing a cystic mass inside the oral cavity is a real illustration of a cancerous mass or an inflammatory one? In this regard, we must always consider that any ancient diagnosis of cancer must be related to the anatomical, medical, and scientific knowledge of the society of that time. 

At the same time, we cannot exclude that the lack of many specific references on oral cancer in the medical literature before the 15th century could be attributed to the fact that several etiologic factors which are thought to be of some significance today, did not appear in Europe until the 15th and 16th centuries (tobacco and alcohol, syphilis); in fact, the clinical appearance of oral cancers is so evident and disruptive, especially in the advanced stages, that there is no way they’d go unnoticed by physicians; we think that its signs in the oral cavity would have made recognition easier if it occurred as frequently as today. Despite this, we believed that genetic susceptibility has been a constant risk factor over the centuries and, more probably, the lower prevalence of oral cancer could be attributed to a lower life-expectative in the past, when it was around 30 years of age and cancer, at that time, was a disease of less importance compared to the terrible infectious diseases, especially tuberculosis and syphilis, so many cases of oral cancers would be invisible to contemporary scholars. 

## 5. Conclusions

All these issues are not simple to solve and should serve to remind us to interpret always historical findings with some caution and skepticism. Several descriptions on oral cancers in antiquity we found let we think that this disease might be linked to mechanisms not strictly dependent on environmental risk factors, and this might address future researches on oral cavity treatments towards strategical cellular and molecular techniques.

## Figures and Tables

**Table 1:** Overview of the main steps of the knowledge about oral cancer and its therapy.

Historical Timeline on Oral Cancer	
**Chronology**	Main Events
**3000–1600 BC**	Edwin Smith and Ebers’ Egyptian papyri descriptions of cancers
**1000 BC**	The encyclopedic Sanskrit medical text, Sushruta Samhita, describes different head and neck cancers.
**6th century BC**	Pythagorean Alcmaeon’s of Croton studies on the oral sensory system in “Περι Φυσεως” (About Nature).
**5th–4th century BC**	Hippocrates’ work “Ιπποκρατικό Σώμα” (Corpus Hippocraticum) first uses the terms cancer/carcinoma (καρκίνος/καρκίνωμα)
**1st–** **2nd century AD**	Galen’s use of the term oncos (όγκοs) to describe the tumor in the treatise “Παρὰ φύσιν ὄγκοι” (On Tumours against Nature), describing also pharmacological and surgical treatment. The Roman author Celsus’ medical encyclopaedia ‘‘De Medicina” describes malignant growths.
**5th–** **14th century AD**	Greek Byzantine and later Arab’s medical texts with detailed descriptions of head/neck and oral cancers (Byzantine diagnostic and therapeutic procedures took place in *Ξενώνες* (Xenones=Hostels), precursors of modern hospitals).
**15th–** **17th century AD**	Andreas Vesalius writes one of the most influential books on anatomical studies, “De humani corporis fabrica” (On the Human Body’s Factory). Introduction of tobacco in the Western World The surgeon P. Marchetti performs the first glossectomy for tongue cancer.
**17th–19th century**	Infectious theories about origin of cancer. First hospital for cancer patients in Rheims, France
**19th century**	Introduction of general anaesthesia in 1846 allowed increasing cancer excisions with the development of many surgical access routes for oral cancer. Beginning of the microscopic era and of the surgical biopsies for diagnostic purposes.
**1885**	Henry T. Butlin, head and neck surgeon, describes in his book “Diseases of the Tongue” an even more radical dissection of the primary tumor “*en bloc*” with surroundings cervical nodes.
**1905–1906**	G.W. Crile (1864-1943) publishes two papers describing a systematic and radical approach to “*en bloc*” dissections.
**1938–1958**	Hayes Martin’s team carries out 1,450 radical neck dissections on patients with oral cancers and cervical metastases.
**1968**	Osvaldo Suarez proposes a ‘‘functional or modified neck dissection’’ preserving the accessory nerves and their functions.
**1990**	Shah et al. demonstrate that the risk of metastasis in nodes of 1,801 patients in levels IV and V was only 9% and 2%, so they concluded that their radical removal in not indispensable in N0 neck cancers.
**2002**	The American Head and Neck Society standardizes neck dissection terminology and techniques, dividing neck nodes in a 6-level classification system.
**21th century**	Even more super-selective nodes dissections in early tumours with multi-integrated pharmacological therapies and reconstructive surgery.

**Table 2:** Summary of established and presumed risk factors for Oral Cancer.

Precursor Conditions	Environmental Factors	Genetic Factors
Infections: HPV, EBV, HIV, *Treponema pallidum* and others (chronic Candidiasis ?)	Lifestyle (e.g., alcohol abuse, distilling cider, tobacco smoking/chewing, Betel quid or guṭkha chewing, marijuana (?), poor dental and oral hygiene)	Fanconi’s anemia
Chronic mouth’s irritation (aggressive mouthwashes, faulty dental prostheses, periodontal disease, gastro-esophageal reflux)	Low socio-economic status (poor or no access to oral health care facilities)	Hereditary genodermatoses (dyskeratosis congenita, xeroderma pigmentosum, scleroderma)
Immune suppression and immune disorders (i.e., trans-planted patients, due to the chronic inflammatory state associated with graft-versus host disease (GVHD)	Industrial pollution or occupational exposures (sulfuric acid, asbestos, formaldehyde, pyrene, methyl pyrene, leather and textile industries workers)	Plummer-Vinson (aka Patterson-Brown-Kelly) syndrome
	Dietary factors (deficiencies of vitamins A, E, B complex, zinc, low intake of fruit and vegetables, especially carrots, fresh tomatoes, and green peppers, manipulated aliments as fried foods)	Genetic polymorphisms of genes coding for enzymes (i.e., P450 and XMEs)
	Radiation exposure (UV-A, Ionizing radiation/radiation therapy)	Diabetes

**Table 3:** The most important viruses associated with oral cancers and their molecular effects in the host.

Most Important Viruses Associated to Oral Cancers	
Virus	Host Events
**EBV** (Epstein Barr Virus)	It stimulates B lymphocytes proliferation and LMP1 production → essential for lymphocytes B transformation It doesn’t have a direct role in carcinogenesis, but it is associated with immunodeficiency A synergy with HPV is assumed (however it has not been demonstrated)
**CMV** (Cytomegalovirus)	It has been implicated with other Herpesviruses in the etiology of several human carcinomas
**HPV** (Human Papilloma Virus)	It is associated with various types of oral lesions: vulgar wart (HPV-4), papillomas (HPV-11), vulgar warts in HIV+ pts (HPV-7), acuminate condylomata and leukoplakia (HPV-6) and squamous cell carcinoma (HPV-16 >98% is associated and HPV-18)
**HSV-1** (Herpes Simplex Virus type 1)	It causes oral carcinoma only if associated with TAR (tobacco associated residues): TAR molecules block the synthesis of DNA polymerase, thymidine kinases, γ proteins → interference with viral shedding → increase of infected cell α-proteins (ICP4 and ICP27) It can directly cause oral cancer or it can be HPV cofactor

## Supplementary Materials

The following are available online at https://www.mdpi.com/1660-4601/17/9/3168/s1.
