# Supplementary material for: Oral Cancer: A Historical Review
Source: Int J Environ Res Public Health. 2020 May 2;17(9):3168. doi: 10.3390/ijerph17093168 (PMC7246763; doi:10.3390/ijerph17093168)
Supplement: Supplementary file 1 [file ijerph-17-03168-s001.pdf]

Supplementary Materials: Oral Cancer: A Historical Review

Synopsis of the original ancient Greek texts and their translation

1. Hippocrates, Hippocratic corpus, *"Αφορισμούς"*(The Aphorisms; chap.7,87):

"…ὁκόσα φάρμακα οὐκ ἰῆται, σίδηρος ἰῆται· ὅσα σίδηρος οὐκ ἰῆται, πῦρ ἰῆται· ὅσα δὲ πῦρ οὐκ ἰῆται, ταῦτα χρὴ νομίζειν ἀνίατα…"

2. Oribasius of Pergamum (Ὀρειβάσιος ο Περγαμηνός) in *"Ιατρικαί Συναγωγαί"* (Medical Collections), *Βιβλίον με΄*(Book LX), *Ἐκ τῶν Ρούφου. Περὶ ἀκροχορδόνων καρκινωμάτων (*From Rufus: about warts and carcinomas*)* , *κεφ. 45.11 (*chapter 45.11):

   "…Ὁ δὲ Ξενοφῶν ἐν τῷ περὶ τῶν καρκινωμάτων εἶδός τί ἀκροχορδόνρος ἱστορεῖ κακόηθες καὶ καρκινῶδες, οὕτω γράφων· Προσαγορεύεται δὲ καρκὶνωμα, καὶ ὅταν ἔκφυσις γὲνηται ἔκ τινός τῶν τοῦ σώματος τόπων, τήν μὲν αὔξησιν είς τό έκτός ποιουμὲνη παραπλησίως ταῖς ἀκροχορδόσι καὶ τοῖς θύμοις, τό δ' εἶδός ἐμφερής ἀκροχόνδρι, μελαντὲρα μὲν καὶ τραχυτὲρα,αδροτὲρα δὲ καί στρογγυλωτὲρα, προσομοιοτὲρα ἤ θύμῳ ἤ ἰχθύον τοῖς ὀφθαλμοῖς καλουμὲνοις ἤ μόρῳ ἐνώμῳ ἤ πὲπονι ἤ ἄλλῳ τινί τοιούτο......συμβαίνει δὲ καὶ τούτων τῶν καρκινωμάτων ἕκαστον τοῖς μὲν μεῖζον γίνεσθαι, τοῖς δ' ἔλασον, καί τοῖς μὲν πλείστοις ἁπλοῦν εἶναι, τοῖς δὲ δισχιδὲς καὶ τρισχιδὲς, καὶ τοῖς μὲν πλείστοις διαμὲνειν ἐπί τοῦ αυτοῦ μεγὲθους, ὃ ἐν τοῖς πρώτοις χρόνοις σχῇ, ἐνίοις δὲ μεῖζον γίνεσθαι, καὶ τισί μὲν τοῦτο βραδύτερον ποιεῖν, τισί δὲ θᾶσσον, καὶ μάλιστα ἐν ταῖς καχεξίαις, καὶ τοῖς μὲν πλείστοις παρὲχειν πόνον, ἐνίοις δὲ καὶ μή παρὲχειν, μάλιστα οἷς ἄν μὲγα γὲνηται παντελῶς γίνεται δ' ἕκαστον τῶν καρκινωμάτων τούτων καὶ ἐν ἄλλοις μὲν τόποις τού σώματος, μάλιστα δὲ τό μὲν τῇ ἀκροχόνδρι ὃμοιον ἐπί χείλους καί ὠτός καί ῥινός ..."

3. Nikolaus Mirepsus Actuarius (*Νικόλαος Μυρεψός Ακτουάριος),* in *"Μέγα Δυναμερόν"*(Great Dynameron or *Medicamentorum Opus"* or *Codex Medicamentarius*):
   "…ξηρίον ωφέλιμον εις δυσοσμίαν ρινός… …και προς καρκινώματα· έχει φλοιού ωών, φλοιού αμυγδάλων, χρυσοβάλου κεκαυμένου, οστέων φοινίκων κεκαυμένων…"
